# Caffeine-Derived Noble Carbons as Ball Milling-Resistant Cathode Materials for Lithium-Ion Capacitors

**DOI:** 10.1021/acsami.1c06013

**Published:** 2021-06-15

**Authors:** Ivan K. Ilic, Enrico Lepre, Nieves López-Salas

**Affiliations:** Colloid Chemistry Department, Max Planck Institute of Colloids and Interfaces, Am Mühlenberg 1, Potsdam 14476, Germany

**Keywords:** ball milling, Li-ion capacitors, caffeine, noble carbons, sustainability

## Abstract

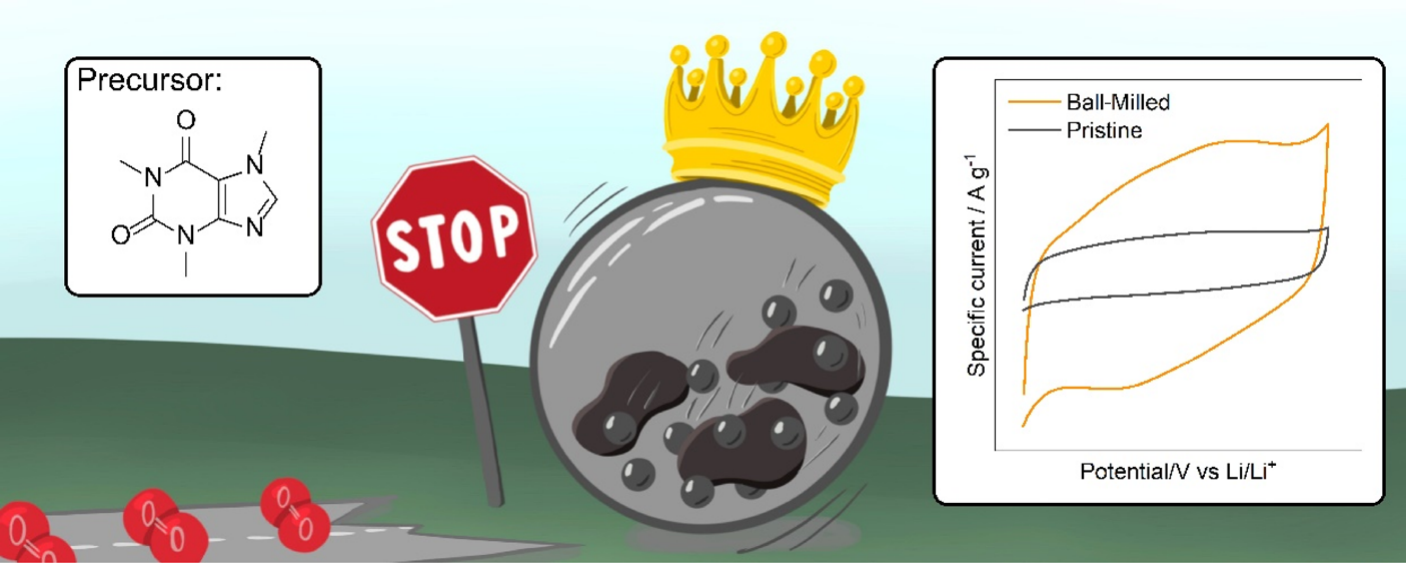

Energy consumption is a growing phenomenon in our society causing many negative effects such as global warming. There is a need for the development of new sustainable materials for energy storage. Carbons are materials derivable from biowaste that can rather easily store energy due to their high conductivity and surface area. However, their large-scale processing is challenging as derived materials can be rather heterogeneous and homogenization requires ball milling, a process that can damage carbons in the process of oxidation. Herein, we have prepared caffeine-derived noble nitrogen-doped carbon that withstands the ball milling process without significant oxidation. Additionally, it performs extraordinarily as a cathode material for lithium-ion capacitors, making it an attractive biowaste-derived alternative to commercial heavy metal cathodes.

## 1 Introduction

Global warming is a man-made phenomenon caused by the excessive production of greenhouse gases. The biggest share of greenhouse gases is carbon dioxide generated during the combustion of fossil fuels. There is an ongoing attempt to replace fossil fuels with carbon-neutral sources, mostly producing electrical energy from sun, wind, and hydropower. However, storage of electrical energy is not trivial and special devices for reversible electrical energy storage (i.e., EES devices) are needed.

The two main types of EES devices are secondary batteries and capacitors. Secondary batteries utilize two different redox reactions occurring on the opposite electrodes for EES, while capacitors utilize high surface area conductive networks for the electrostatic adsorption of ions on the surface.^1,2^ While secondary batteries exhibit high energy densities, capacitors show high power densities. Less than two decades ago, ion capacitors, a new type of hybrid EES device merging the advantages of batteries and capacitors, started to be developed.^3−6^ Ion capacitors utilize one battery-type electrode and one capacitor-type electrode to deliver a device with energy densities higher than capacitors and power densities higher than batteries.^7^ Hybrid lithium capacitors are of special interest as existing lithium-ion battery technologies, such as anodes, can be directly applied.

A common cathode material for lithium-ion capacitors is activated carbon due to its low price, good conductivity, high surface area, and synthesizability from biowaste.^8,9^ Furthermore, nitrogen doping has been proven to improve the electroactivity of hybrid ion capacitors,^10,11^ making highly N-doped materials an interesting choice for the synthesis of the new generation of lithium-ion hybrid capacitors. However, activated carbons and related materials are usually obtained as powders requiring the processing to prepare the electrodes. The preparation of electrodes from powdery materials is not a standardized process, and it varies from one research school to another. Though electrode preparation directly affects many of the properties of the final device tested, the process is often overlooked in research papers. Among the different strategies, ball milling using stainless steel jars is a well-established technique implemented in industry at a very large scale due to the fact that stainless steel is a relatively inexpensive as compared to other common ball milling materials such as tungsten carbide and zirconia. In the context of electrode preparation, ball milling is used to prepare homogeneous electrode inks by mixing the active material, a conductive additive, the binder, and the dispersing agent since it can easily be scaled up.^12,13^ However, when ball milling the ink mixtures, they are submitted to harsh oxidation conditions. As a result, the active carbon materials undergo oxidation^14−16^ and end up contaminated mainly with iron coming from the stainless steel balls, as described previously for inorganic compounds.^17,18^ As a result, the ball milling equipment erodes and the performance of the material becomes questionable as it often occurs that the effect of the metallic impurities in the carbonaceous materials is treated as part of the performance of the material.^19,20^ In this context, noble carbons are very promising candidates for effective ball milling. Noble carbons are a new class of carbon-based materials comprising a high resistance upon oxidation.^21^ Noble carbons are obtained upon heat treatment of a series of specific organic precursors that are already very resistant upon oxidation themselves (i.e., ionic liquids, nucleobases, or certain organic dyes).^12,22−25^ Contrary to what occurs when carbonizing a conventional precursor (i.e., glucose), a noble carbon precursor decomposition leads to the formation of new and even more stable bonds to satisfy thermodynamics, which results in materials with low HOMO levels and great stability upon oxidation.^21^ Recently, a noble C_1_N_1_-like material exhibiting large stability upon oxidation derived from guanine, a purine nucleobase, was reported. At high temperatures, the synthesis yielded a CN covalent material comprising less nitrogen (C/N ratio of 7) but still exhibiting high resistance upon oxidation and an ultrahigh surface area, thus standing as an excellent candidate for EES electrode ink preparation *via* ball milling.^24,25^

In this paper, a noble CN material derived from caffeine (CafCN), a xanthine comprising a structure similar to purines, was compared to activated carbon with similar porosity (KC) and its nitrogen-doped analog (N-KC) in terms of electrochemical activity and resistance upon ball milling. The results were critically compared giving a great emphasis on the changes of these carbons as a result of electrode preparation *via* ball milling.

## 2 Materials and Methods

### 2.1 Synthesis of the Materials

All the materials were used without further purification. Caffeine (99%) and cyanamide (98%) were purchased from Sigma-Aldrich. Kuraray was purchased from MTI. Hydrochloric acid was purchased from Merck. ZnCl_2_ was purchased from Alfa Aesar, and NaCl was purchased from Fischer Chemicals. 1-Methyl-2-pyrrolidinone 99% (NMP) was purchased from Alfa Aesar and polyvinylidene difluoride (PVDF) was purchased from MPI Corporation.

#### 2.1.1 Synthesis of CafNC

In a normal procedure, caffeine (1 g) was mixed with 20 g of NaCl/ZnCl_2_ (1/1 wt %) by grinding in an Agate mortar. The solid mixture was submitted to carbonization at 700 °C (2 h, 1 °C min^–1^). After spontaneous cooling down, the system was washed with HCl (1 M, 400 mL) 3 times at room temperature. After the last washing, the material was dried at 70° at atmospheric pressure for 6 h and then at 150 °C in a vacuum overnight. The final product was obtained as a black fluffy powder, and it was obtained with 30% yield.

#### 2.1.2 Synthesis of N-KC

Cyanamide (2 g) was solubilized in 2 mL of water. Commercial Kuraray (1 g) was soaked in cyanamide solution and stirred for 6 h. After stirring, water was evaporated at 60 °C for 12 h. The dried solid mixture was then submitted to heat treatment at 800 °C (4 h, 1 °C min^–1^). After spontaneous cooling down, the product was obtained as a black fluffy powder.

#### 2.1.3 Ball Milling

To compare different carbons regarding their stability to ball milling, the samples (54 mg) were put in stainless steel jars (12.5 mL equipped with five stainless steel balls with a diameter of 1 cm) and ground for 50 min at 650 rpm in a planetary ball mill (Retsch PM 100).

### 2.2 Characterization

Powder X-ray diffraction patterns were recorded with a Bruker D8 Advance instrument with Cu-Kα as a radiation source (λ = 0.154 nm). A NaI scintillation counter detector was used to observe X-ray diffractions. The patterns were analyzed in a range between 5 and 70° for 2θ with steps of 0.05° and 2 s.

Thermogravimetric analysis was carried out in synthetic air as a gas carrier (20 cm^3^ min^–1^) and with a heating rate of 10 K min^–1^ in a Pt crucible. In a typical analysis, 10 mg of samples was weighed with a Thermo Microbalance TG 209 F1 Libra (Netzsch Selb, Germany) while increasing the temperature.

A PerkinElmer ICP-OES Optima 8000 was used to perform inductively coupled plasma optical emission spectroscopy (ICP-OES). In a standard procedure, 10 mg of samples was dissolved in 500 μL of a 1:3 HNO_3_:HCl mixture before the analysis. The heterogeneous mixture was kept at room temperature for 12 h and at 96 °C for 1 h. After spontaneous cooling down, the samples were diluted in ultrapure water to 10 mL and filtrated after the measurement. Four calibration standard solutions per element were used to obtain the curve.

Scanning electron microscopy (SEM) was recorded on a LEO 15550-Gemini instrument. Before the analysis, the samples were sputtered with a ca. 10 nm layer of an 80% gold and 20% platinum mixture. Energy-dispersive X-ray (EDX) spectroscopy was run using a couple of EDX analyzers (Oxford Instruments). An EM 102 Zeiss instrument operating at 120 kV was used to record transmission electron microscopy micrographs (TEM). The samples were prepared on a copper grid by drop-casting sample dispersions in ethanol.

Nitrogen adsorption and desorption isotherms at 77 K were analyzed by a Quantachrome Quadrasorb SI apparatus. A 3P Instruments Masterprep degassing machine was used to degas the samples at 150 °C under vacuum (0.5 Torr) for 20 h before the measurements. The Brunauer–Emmett–Teller (BET) method was used to calculate the specific surface area from adsorption branch data (*P*/*P*_0_ < 0.2). The total pore volume (*V*_T_) was calculated from the amount of gas adsorbed at *P*/*P*_0_ = 0.995. Pore size distributions were calculated by the nitrogen adsorption branch using the quenched solid density functional theory (QSDFT) model.

### 2.3 Electrochemical Characterization of the Materials

Electrodes were prepared by taking 42 mg of the respective carbon material and ball milling it along with 12 mg of Super P carbon in stainless steel jars (12.5 mL equipped with five stainless steel balls with a diameter of 1 cm) for 50 min at 650 rpm in a planetary ball mill (Retsch PM 100). After the initial grinding, 1 mL of PVDF solution was added (concentration, 6 mg mL^–1^) and the prepared sample was further ground for 10 more minutes. The prepared ink (40 μL) was dropped on 11 mm carbon paper discs (Spectracarb 2050A-0550) that were dried for 1 h at 80 °C in the air to remove possible volatile impurities. The formed electrodes were dried at 120 °C for 17 h under vacuum. Sample ng-C/CafNC was prepared without initial grinding. Loading of all the electrodes was 2.0–2.5 mg cm^–2^. Furthermore, the exact mass of all the electrodes can be found in Table S4.

All electrochemical measurements were performed in Swagelok-type cells. Lithium foil, Celgard 2325 (13 mm in diameter, 25 μm thick), and 1 M LiPF_6_ in ethylene carbonate and diethyl carbonate (volumetric ratio of 1:1, 100 μL, a solution used as obtained from Sigma-Aldrich) were used as the counter electrode, membrane, and electrolyte, respectively. A circular carbon current collector covered with the active material was used as a working electrode. All Swagelok-type cells were assembled in a glovebox with low water and oxygen levels. Charging-discharging measurements were performed at constant current densities (0.2 A g^–1^ for 100 cycles and 0.8 A g^–1^ for 1000 cycles) or at varying current densities (20 cycles at 0.05 A g^–1^, 10 cycles at 0.10 A g^–1^, 10 cycles at 0.20 A g^–1^, 10 cycles at 0.40 A g^–1^, 10 cycles at 0.80 A g^–1^, and 10 cycles at 0.05 A g^–1^) in triplicate. Cyclic voltammetry was performed by cycling at 5 mV s^–1^ during 30 cycles and at 2, 1, 0.5, 0.2, and 0.1 mV s^–1^ for subsequently one cycle each. In the displayed cycling voltammetry curves, the last cycle at the respective speed is always shown unless mentioned otherwise.

## 3 Results and Discussion

### 3.1 Synthesis and Characterization of Caffeine-Derived Noble Carbon (CafNC)

A porous NC material was prepared from caffeine by submitting the precursor to heat treatment in the presence of a NaCl and ZnCl_2_ (1:1 wt/wt) salt mix as porogen.^12,24,26,27^ In brief, 1 g of caffeine was mixed in a ratio of 1:20 wt/wt with the salt mix and treated at 700 °C in a nitrogen atmosphere. After cooling down, the rest of the salts were washed away using 1 M HCl. The prepared sample will be named hereafter as CafNC. SEM–EDX results (Table 1) show that CafNC is a nitrogen-rich carbonaceous material with a C/N ratio of 3.2.^24^ For comparison purposes, commercial non-noble porous carbon (i.e., Kuraray active carbon, herein KC) and nitrogen-doped carbon KC (herein N-KC) have been used (further details on N-KC preparation can be found in the Supporting Information). While KC shows a C/N ratio equal to 16, after being doped, N-KC shows a C/N ratio of 8.2. Fourier transformed infrared spectroscopy (Figure S1a) was used to evaluate chemical bond differences between the samples. While KC and N-KC show almost no absorption features, CafNC shows bands at 3200, 1576, 1192, and 663 cm^–1^ ascribed to N–H, C=N, C–N, and C–N-heterocycle vibrations of the material, respectively. XRD patterns (Figure S1b) show a broad peak centered at 26° typically ascribed to graphitic stacking. The broad nature of the peak indicates that samples are not well ordered. In the case of KC and N-KC, a second broad peak at 44° ascribed to in-plane carbon atoms is visible.^28^

**Table 1:** Specific Surface Area, Total Pore Volume, and EDX Results

			EDX			
sample	*S*_BET_ (m^2^ g^–1^)	total pore volume (cm^3^ g^–1^)	C (wt %)	N (wt %)	O (wt %)	C/N (at)
CafNC	1496	0.654	70	22	5	4
KC	1672	0.763	80	5	5	18
N-KC	1422	0.635	86	10	4	10

Scanning electron microscopy images of CafNC at large magnification show a rough surface, indicating that the salt melt chosen acted as an effective template to induce a very narrow porous network in the sample (Figure S2). The KC surface is rough as expected due to the characteristic of the commercial sample. Sample N-KC preserves its surface roughness, also indicating that the porous network of KC was not fully clogged by the synthetic strategy chosen to introduce heteroatoms. On the other hand, transmission electron microscopy images suggest the presence of a mesoporous network for samples KC and N-KC whereas a much flatter morphology of CafNC indicates mainly the presence of micropores in the sample.

**Figure 1:**
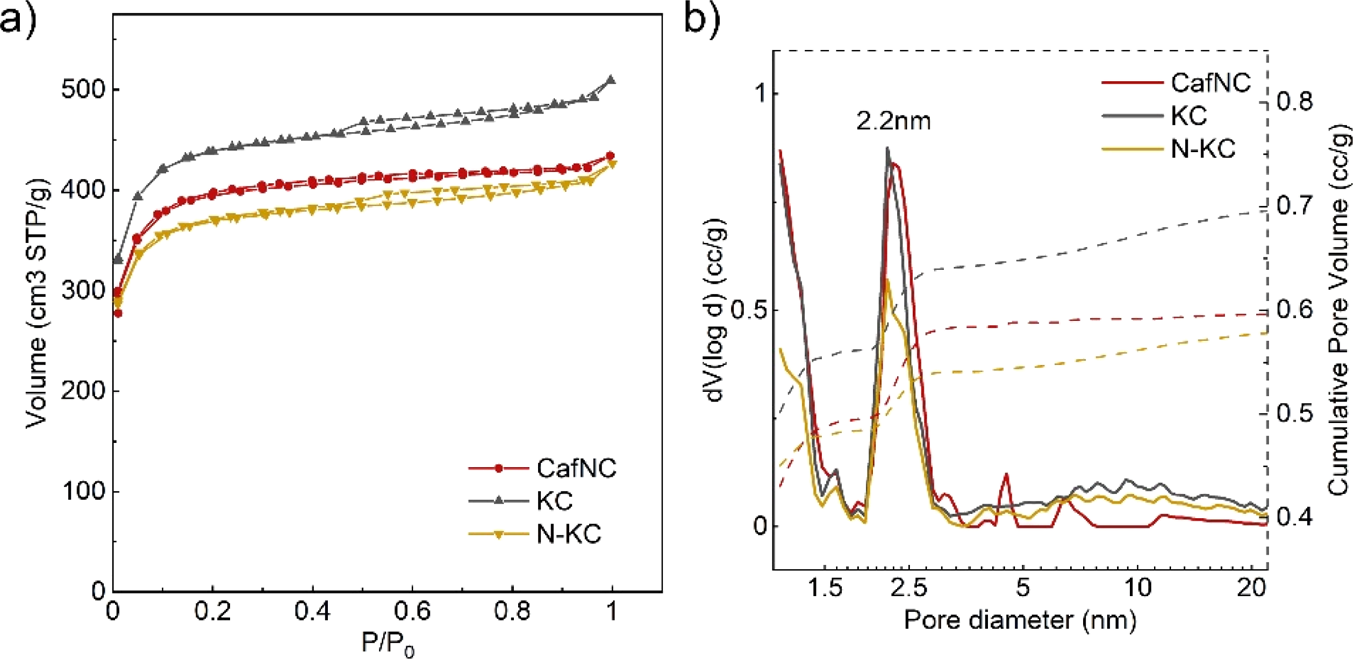
(a) N_2_ adsorption/desorption isotherm at 77 K and (b) pore size distribution and cumulative pore volume calculated by QSDFT from the N_2_ adsorption branch at 77 K of CafNC, KC, and N-KC.

N_2_ adsorption isotherms were used to further evaluate the porous morphology of the materials. KC and N-KC exhibit a combination of type I and type IV N_2_ adsorption isotherms at 77 K indicative of the presence of both mesopores and micropores in the samples (Figure 1a). On the other hand, CafNC shows a type I isotherm with a slight hysteresis at intermediate relative pressures, indicating that the sample is mainly microporous but also shows some mesoporosity. Specific surface areas were calculated using the Brunauer–Emmett–Teller (BET) method. As can be seen in Table 1, the materials show very similar surface areas (i.e., 1496, 1672, and 1422 m^2^ g^–1^ for CafNC, KC, and N-KC, respectively) and total pore volumes. Pore size distribution (PSD) calculated using quenched solid density functional theory (QSDFT) to nitrogen adsorption isotherms shows that all the samples present well-defined mesopores of 2.2 nm in diameter (see Figure 1b).

**Figure 2:**
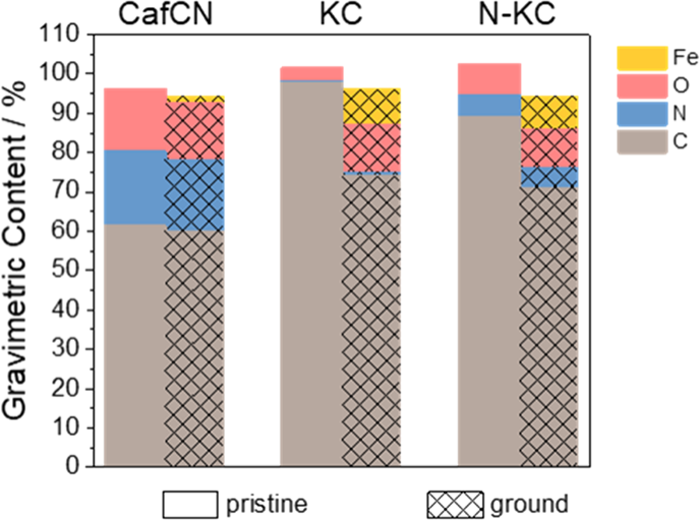
Elemental content of pristine and ground CafNC, KC, and N-KC as determined from combustive elemental analysis (C, N, and O) and ICP-EOS (Fe).

**Figure 3:**
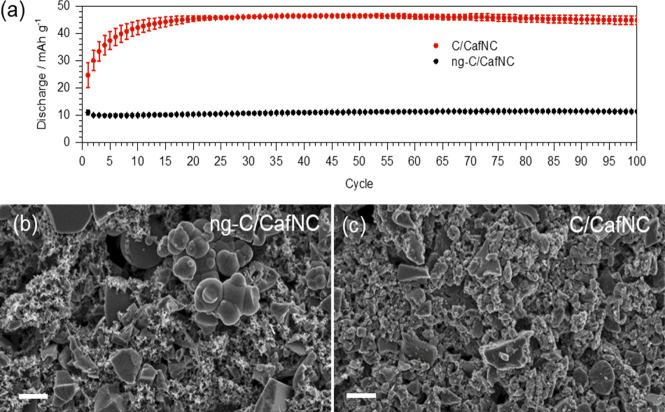
(a) Discharge capacities as calculated from charging-discharging tests of CafNC with (C/CafNC) and without (ng-C/CafNC) prior ball milling. SEM images of ng-C/CafNC (b) and as-prepared C/CafNC (c) electrodes. The white bars represent 2 μm.

Previously, it was reported that non-noble carbons tend to get severely oxidized upon ball milling.^14,15,29^ In the process, they absorb iron from the sample, but no trace of iron particles was observed. It was concluded that iron leakage during ball milling is connected to the oxidation of carbon. CafNC, as representative noble carbon, Kuraray carbon (KC), and nitrogen-doped Kuraray carbon (N-KC) were ball-milled for 50 min and compared. Elemental analysis before and after ball milling for 50 min was run in all three samples (Table S1 and Figure 2). Upon grinding, both KC and N-KC undergo significant oxidation (lower relative carbon content), which is not observed in the case of CafNC. This points to the high oxidative resistance of CafNC as part of the noble carbon family. Thermogravimetric analyses in synthetic air for CafNC, KC, and N-KC (Figure S3) give more information on the type of stability of the samples toward oxidative environments. Interestingly, CafNC shows lower stability of this material when compared to KC and N-KC, which indicates that the oxidation path where the sample undergoes upon ball milling is different from the one occurring upon heat treatment. The differences between thermal oxidation and oxidation upon ball milling made us track iron and chromium contamination upon ball milling by ICP-OES (Figure 3 and Table S2). All the pristine carbons contained insignificant levels of iron contamination, but upon ball milling, KC and N-KC absorb significantly more iron than CafNC. Similar iron absorption by KC (nitrogen content around 1%) and N-KC (nitrogen content around 5%) highlights the independence of metal adsorption from the nitrogen content. The much lower iron uptake of CafCN and much larger resistance upon ball milling oxidation indicate that the oxidation mechanism occurring involves the incorporation of metals from the grinding balls. Interestingly, noble CN materials resist that process much better than conventional carbons. To prove that the resistance does not only correlate to the nitrogen content of the sample, a sample identical to CafCN but treated at 800 °C (herein called CafCN800) was prepared and its composition was analyzed before and after 50 min of ball milling. The results summarized in Table S3 show that sample CafCN800 has a very similar composition to N-KC; however, its resistance toward oxidation is similar to that of CafCN, i.e., nor oxygen neither iron content increase upon ball milling.

### 3.2 Enhanced Performance of Ball-Milled CafNC

The electrochemical performance of CafNC was tested after its incorporation in electrodes. CafNC powder was mixed with a conductive additive and ground for 50 min. Afterward, 1 mL of NMP with a dispersed binder was added and the mixture was further milled for 10 min to assure good dispersion of the ink (details of the procedure are given in the Supporting Information). Upon drying, a sample denoted as C/CafNC was obtained. The importance of ball milling before ink preparation was assessed by the electrochemical test of the ink prepared without prior ball milling (ng-C/CafNC). Performances of C/CafNC and ng-C/CafNC were evaluated *via* galvanostatic charging-discharging measurements at 0.2 A g^–1^ during 100 cycles (Figure 3a and Figure S4). Alike non-noble carbons, ball-milled CafNC shows superior electrochemical performance to the non-ball-milled ones.^14,30^

A better understanding of the differences between the pre-ground and directly prepared carbon ink was achieved by analyzing the samples using scanning electron microscopy (SEM) (Figure 3b,c). SEM micrographs show how C/CafNC exhibits much smaller particles than r-C/CafNC, resulting in better contact between the particles of CafNC and the conductive additive. Additionally, the homogeneity of the sample was evaluated *via* energy-dispersive X-ray spectroscopy (EDX), and contrary to the high nitrogen content of CafNC, the conductive additive has virtually no nitrogen. EDX mapping (Figure S5) shows the homogeneous distribution of all the elements in the ball-milled sample; however, it shows the heterogeneous distribution of nitrogen without prior ball milling, suggesting an inhomogeneous sample with regions rich with Super P and CafCN.

The performance of the three carbons as electrodes in lithium-ion supercapacitors was analyzed (Figure S6). While KC and N-KC perform similarly, CafNC performs significantly better, more than twice as good upon 50 cycles. This can be ascribed to the resistance of CafNC to oxidative damage upon sample preparation and its larger nitrogen content. For instance, it is well known that a high nitrogen content increases the performance of supercapacitors due to electrostatic stabilization of adsorbed ions^31^ as all three pristine carbons have otherwise similar porosities, as measured by nitrogen (Figure 1a).

### 3.3 Detailed Investigation of Electrochemical Performance of CafNC

**Figure 4:**
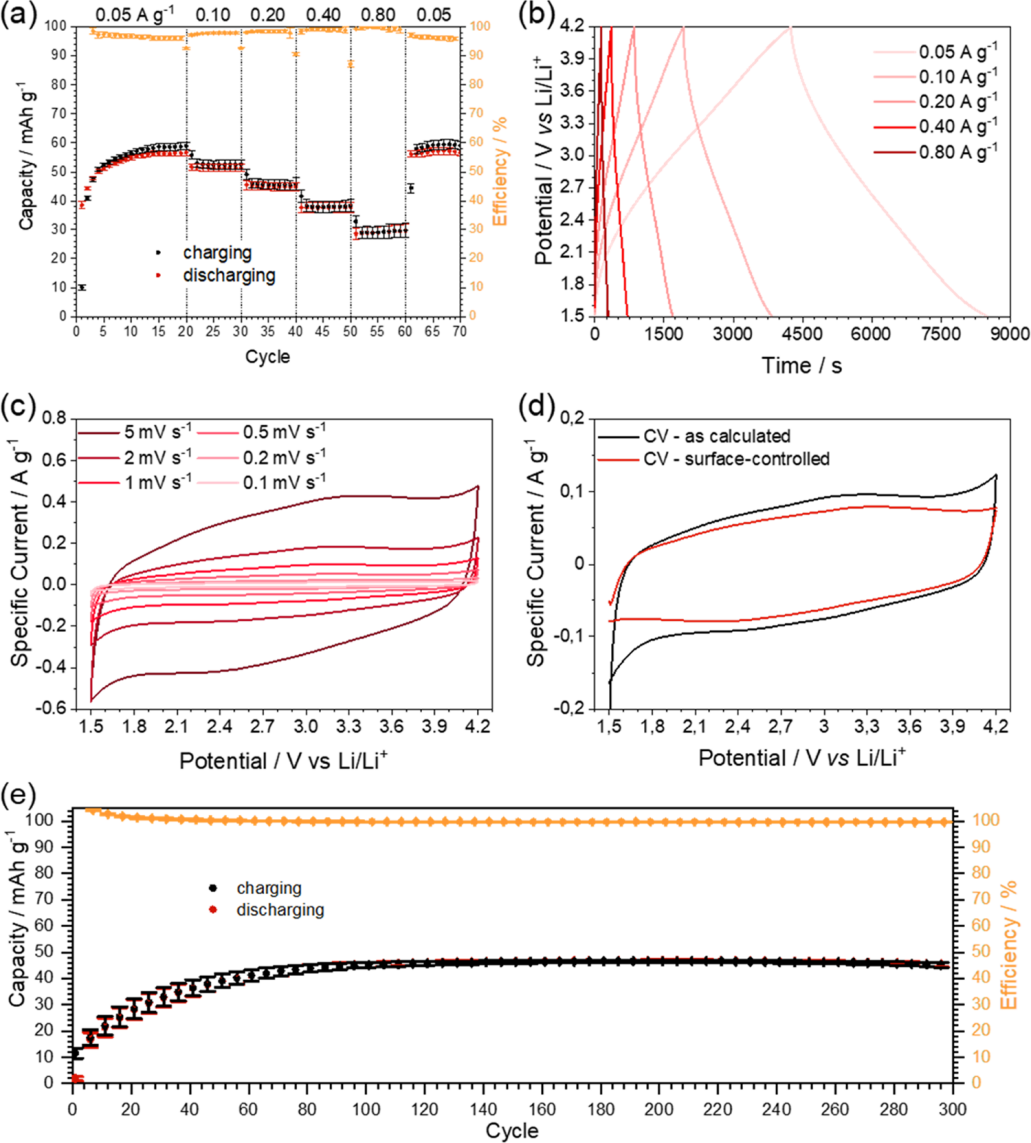
Detailed study of the electrochemical performance of C/CafNC. (a) Charging-discharging tests at varying current densities. (b) Charging-discharging profiles at different current densities. (c) Cyclic voltammetry of C/CafNC at varying scan rates. (d) Calculated surface-controlled contributions to capacity. (e) Charging-discharging test at 0.8 A g^–1^ for 300 cycles.

Electrochemical performance of C/CafNC was studied in depth *via* charging-discharging tests at varying current densities, for 300 cycles at a constant current density of 0.8 A g^–1^, and *via* cyclic voltammetry at varying scan rates (Figure 4). Samples tested *via* charging-discharging tests at different current densities were first cycled for 20 cycles at 0.05 A g^–1^ until no significant change in the capacity was observed. Subsequently, the current density was increased to 0.1, 0.2, 0.4, and 0.8 A g^–1^ for 10 cycles each, only to be reduced to the beginning value for the last 10 cycles (Figure 4a). At a current density of 0.05 A g^–1^, the maximum observed discharge capacities are around 55 mA g^–1^, and as expected, they are reduced upon the increase in current densities. However, no significant reduction has been observed as discharge capacities of around 30 mAh g^–1^ are visible at a high current density of 0.8 A g^–1^. Upon the decrease in current density to the original value of 0.05 A g^–1^, the capacities increase reaching similar values as in the first 20 cycles, pointing that no damage was done to the samples at higher current densities. Charging-discharging profiles are distorted triangles as expected for hybrid lithium capacitors (Figure 4b).^7^ Additionally, they show that both charging and discharging are quick so that a full cycle occurs in less than 5 min at 0.8 A g^–1^.

Cyclic voltammetry experiments were performed at varying scan rates. First, the cell was cycled at 5 mV s^–1^ for 30 cycles, allowing for the cyclic voltammogram to stabilize (Figure S7). At changing scan rates, no significant change in the shape of the curve was observed (Figure 4c) and the distorted rectangular shape, appearing as peaks, is characteristic for hybrid lithium capacitors. Distortion can be ascribed to the limited accessibility of cations into the pores at the potentials below the open-circuit voltage (around 3.2 V, as calculated from Figure S8).^32^ From experiments, at varying scan rates, surface- and diffusion-controlled contribution to the capacity were determined according to the model well described in the previous studies.^33,34^ As expected for a capacitor, an almost purely surface-controlled reaction was observed (Figure 4d). Details of the calculation can be found in the Supporting Information, assessment of the model can be found in Figure S9, and the comparison of the calculated and measured cyclic voltammograms can be found in Figure S10.

Last, the sample was cycled at 0.8 A g^–1^ for 300 cycles (Figure 4e). The capacity first reaches, due to slow wetting of more and more of the carbon’s surface, a capacity of around 45 mAh g^–1^, retaining the said capacity without significant loss after 200 more cycles. The extraordinary performance of the material can be ascribed to a high nitrogen content and ball-milling resistivity. As the material is biowaste-derived, it holds great potential for commercial applications.

## 4 Conclusions

Noble carbon, a class of nitrogen-rich carbonaceous material characterized by its resistance to oxidation, derived from caffeine was prepared and characterized. Additionally, it was used as a model noble carbon material to investigate its resistivity to oxidation during ball milling and compared to non-noble carbon and nitrogen-doped non-noble carbon with similar porosity. Non-noble carbon withstands not only a much lower oxidation but also a much lower uptake of iron from the milling equipment. Last, such carbon performs much better than its non-noble counterpart does as a lithium-hybrid capacitor, achieving extraordinary performance ascribed to its processability in the ball mill and high nitrogen content.

## Supporting Information Available

FTIR transmittance spectra, powder XRD diffraction patterns, SEM and TEM micrographs and thermogravimetric analysis in synthetic air of CafNC, KC, and N-KC; the 50th cycle charging and discharging curve of CafNC with and without prior ball milling at 0.2 A g^–1^; the 50th cycle charging and discharging curve of CafCN, KC, and N-KC at 0.2 A g^–1^; SEM and EDX mapping (carbon, nitrogen, and oxygen) of the electrodes as prepared with and without prior ball milling; integrated cyclic voltammograms at 5 mV s^–1^; open-circuit voltage of the C/CafNC cell before the cyclic voltammetry test; the assessment of the model used for surface-controlled contribution evaluation and cyclic voltammogram at 1 mV s^–1^ as calculated and as measured; elemental chemical analysis of samples before and after the ball milling grinding process; a summary of ICP analysis before and after the ball milling grinding procedure; the elemental chemical analysis of CafNC800 before and after ball milling; and the masses of all the electrodes used in the preparation of this manuscript (PDF)
