# Supplementary material for: Caffeine-Derived Noble Carbons as Ball Milling-Resistant Cathode Materials for Lithium-Ion Capacitors
Source: ACS Appl Mater Interfaces. 2021 Jun 15;13(25):29612–8. doi: 10.1021/acsami.1c06013 (PMC8251692; doi:10.1021/acsami.1c06013)
Supplement: Supplementary file 1 — am1c06013_si_001.pdf [file am1c06013_si_001.pdf]

Supporting Information

Caffeine derived noble carbons as ball milling resistant cathode materials for lithium ion-capacitors

Ivan K. Ilic,[a,¥] Enrico Lepre,[a,¥] Nieves Lopez Salas [a*]

[a] Colloid Chemistry Department, Max Planck Institute of Colloids and Interfaces, Am Mühlenberg 1, 14476, Potsdam e-mail: nieves.lopezsalas@mpikg.mpg.de

[¥] I. K. Ilic and E. Lepre contributed equally to this work.

Characterization of the materials

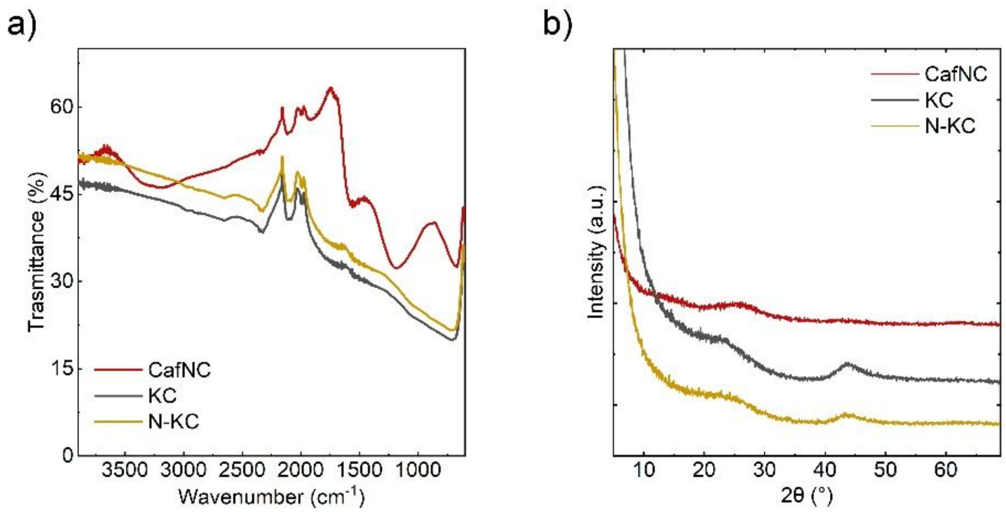

Figure S1. (a) FTIR transmittance spectra and (b) powder XRD diffraction patterns of CafNC, KC, and N-KC.

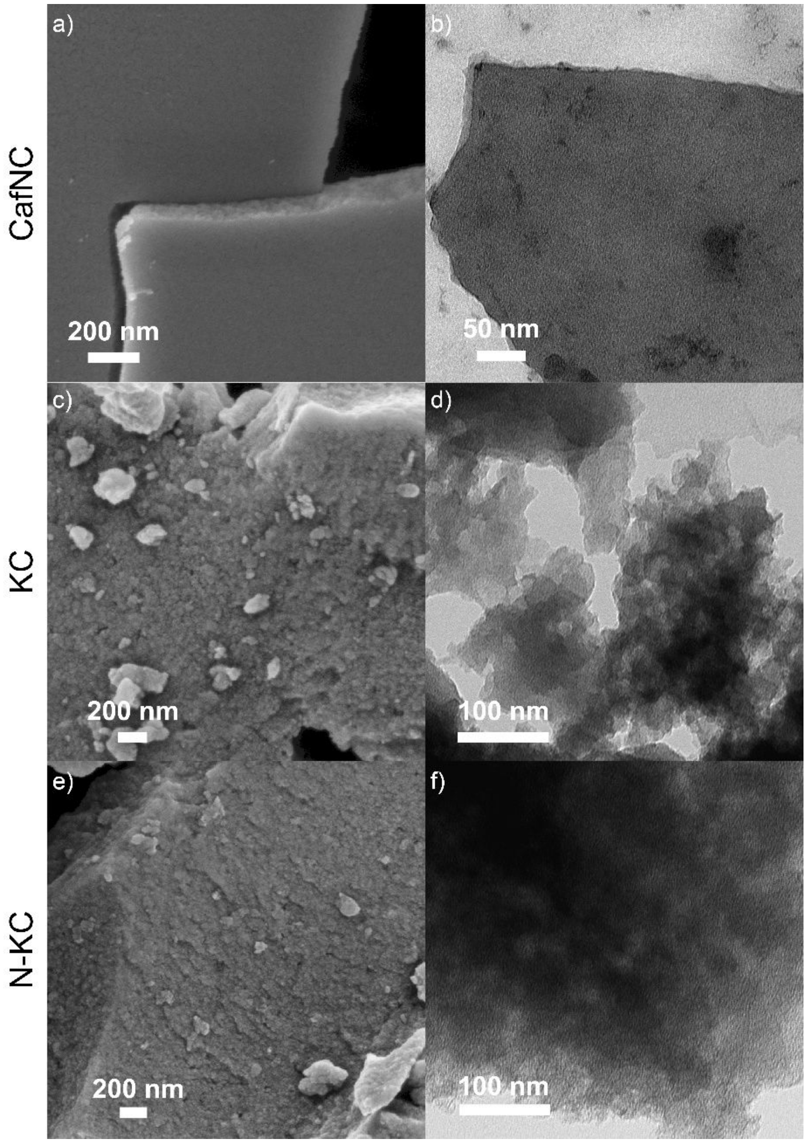

Figure S2. (a, c, e) SEM and (b, d, f) TEM micrographs of CafNC, KC, and N-KC

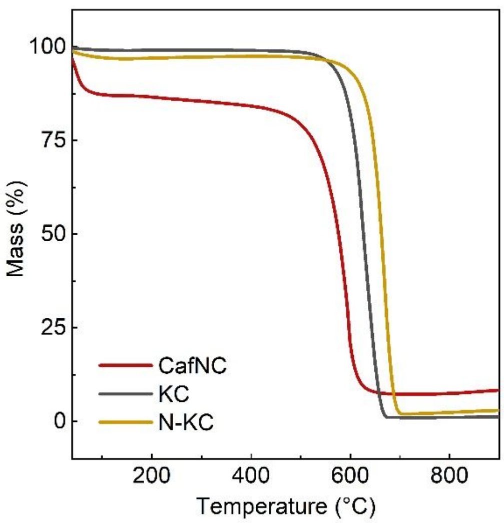

Figure S3. Thermogravimetric analysis in synthetic air for CafNC, KC, and N-KC.

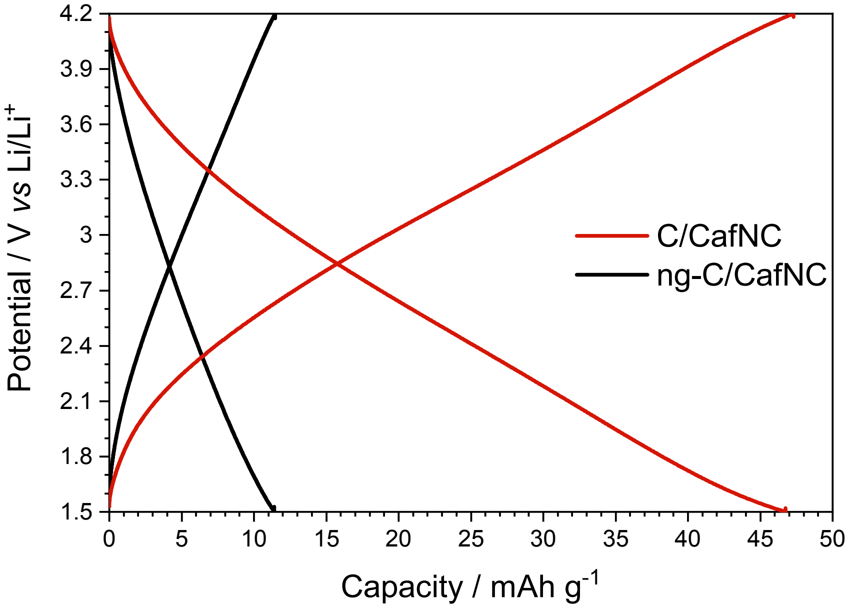

Figure S4. 50th cycle charging and discharging curve of CafNC with and without prior ball milling at 0.2 A g^{-1}.

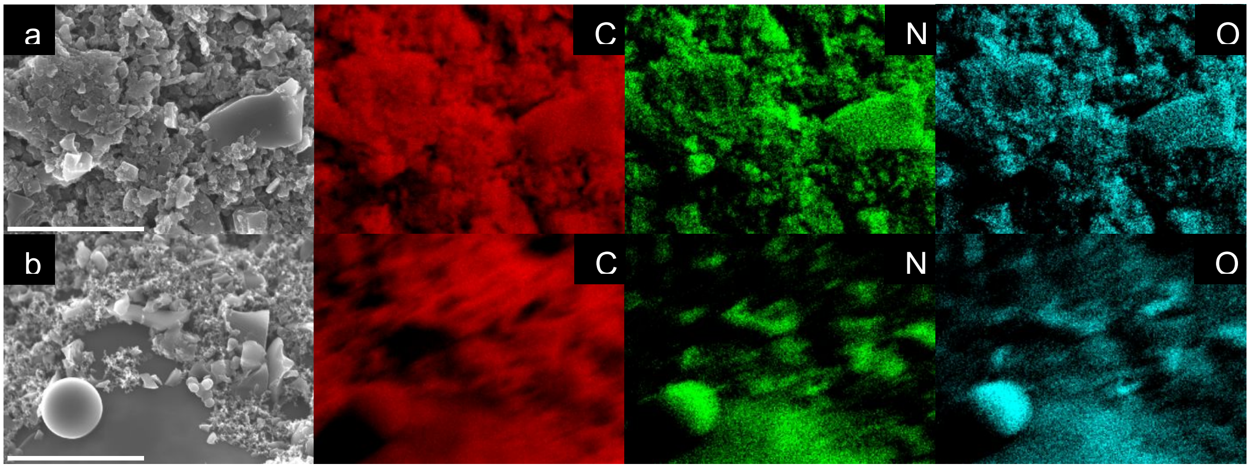

Figure S5. SEM and EDX mapping (carbon, nitrogen, and oxygen) of the electrodes as prepared with (a) and without (b) prior ball milling. The white scale bar equals 10 µm.

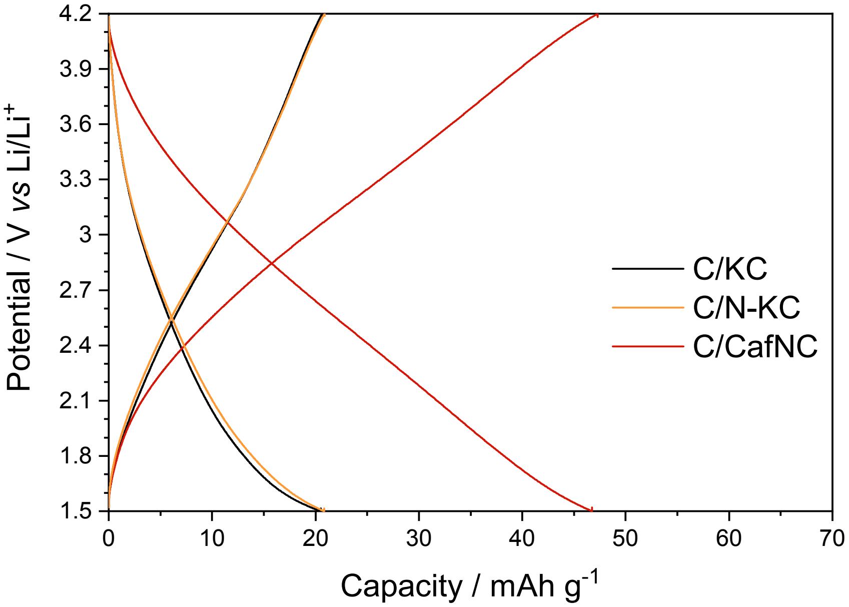

Figure S6. 50th cycle charging and discharging curve of CafCN, KC, and N-KC at 0.2 A g^{-1}.

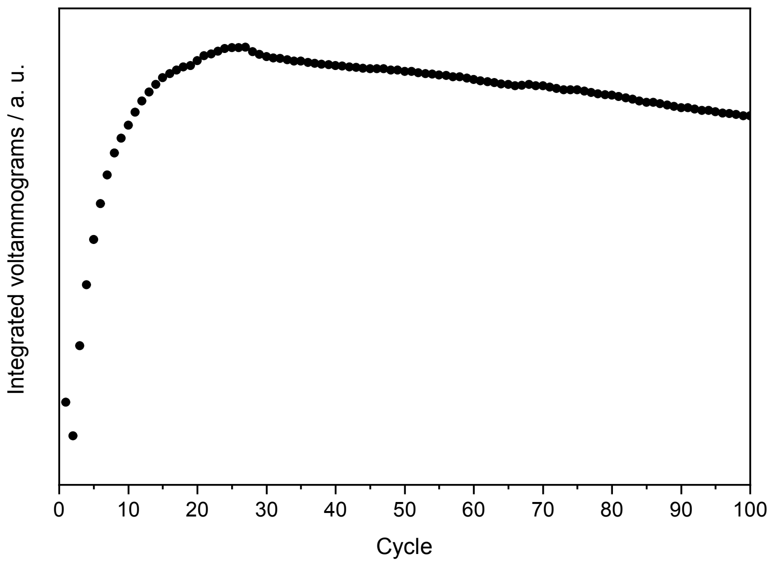

Figure S7. Integrated cyclic voltammograms at 5 mV s^{-1}.

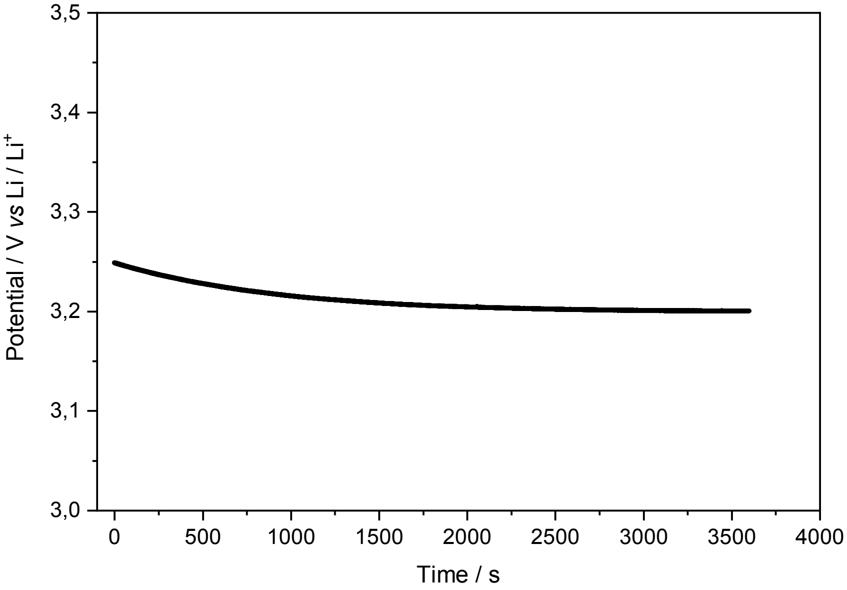

Figure S8. Open circuit voltage of the C/CafNC cell before cyclic voltammetry test.

Electrochemical phenomenon can be either surface- or diffusion-controlled. Current that arrises as a consequence of surface-controlled reactions is proportional to the scan rate, while the diffusion-controlled controlled reactions result in current proportional to the square root of the scan rate and therefore the overall current can be written as:

$$I(v) = k_S v + k_D \sqrt{v} \qquad \text{(Eq. 1)}$$

where $I(v)$ is the total current at a specified scan rate, v is the scan rate and k_S and k_D are constants associated with the surface- and diffusion-controled reacitons respectively. From the linearization of Eq. 1 one obtains:

$$\frac{I(v)}{\sqrt{v}} = k_S \sqrt{v} + k_D \qquad \text{(Eq. 2)}$$

one can calculate k_S and k_D constants at different potentials and subsequently contributions of surface- and diffusion-controlled reactions at the chosen potential.

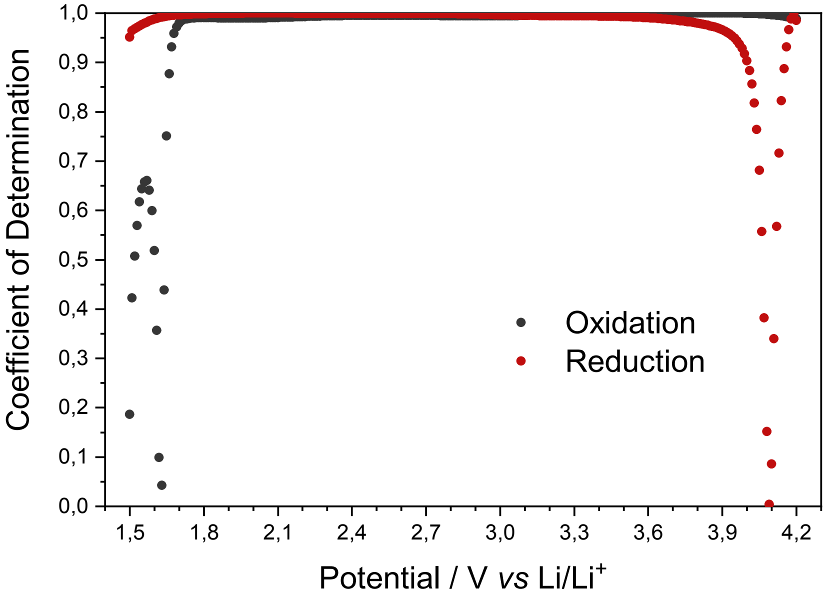

Figure S9. Assessment of the model used for ssurface-controlled contribution evaluation

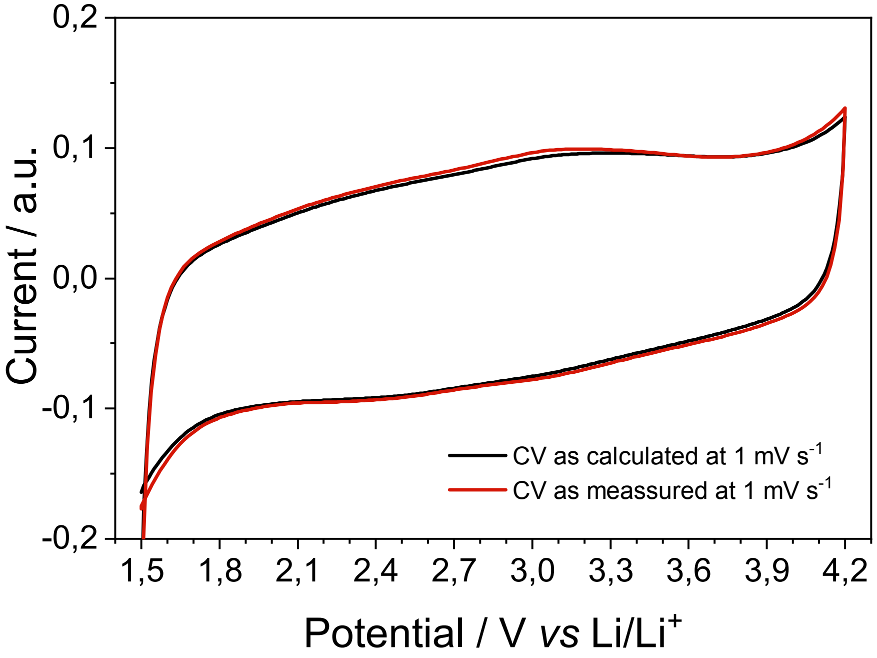

Figure S10. Cyclic voltammogram at 1 mV s^{-1} as calculated and as measured.

Table S1. Elemental chemical analysis of samples before and after ball milling grinding process.

Name	C [w%]	N [w%]	H [w%]	O [w%]
KURRAY	97.86	0.29	0.47	3.54
grinded KC	73.99	1.09	0.79	12.09
N-KURRAY	89.17	5.65	0.39	7.62
grinded N-KC	71.17	4.96	0.64	10.03
CafNC	61.37	18.95	2.26	16.02
grinded CAfNC	60.11	18.01	2.20	14.45

Table S2. Summary of ICP analysis before and after ball milling grinding procedure.

Sample	Fe mg/g	Stdev	Zn mg/g	Stdev	Cr mg/g	Stdev
CafNC	0.201	0.005	13.8	0.130	0.016	0.001
gCafNC	21.3	0.1	16.2	0.2	2.60	0.07
Kurray	0.022	0.001	0.107	0.002	0.002	0.000
gKurray	95.2	1.1	0.121	0.001	11.3	0.080
N-Kurray	0.072	0.001	0.634	0.004	0.001	0.001
gN-Kurray	82.4	0.8	0.46	0.01	11.6	0.1

Table S3. Elemental chemical analysis of CafNC800 before and after ball milling.

Sample	C [wt.%][a]	N [wt.%][a]	H [wt.%][a]	O [%][a]	Fe [%][b]
CafNC800	62.67	12.65	1.80	13.48	0.02
Grinded CafNC800	60.80	12.69	1.86	15.74	0.5

[a] Data obtained by elemental analysis, [b] data obtained by ICP-OES

Table S4. Masses of all the electrodes used in the preparation of this manuscript. As most of the experiments were done in triplicate, masses of all the electrodes are reported here. All the masses are given in miligrams.

Sample	Measurement	m_1	m_2	m_3
C/CafCN	CD@0.2 A g^{-1}	2.32	2.43	2.28
ng-C/CafCN	CD@0.2 A g^{-1}	2.76	2.50	2.76
C/CafCN	CD@varying	2.35	2.38	2.38
C/CafCN	CV@varying, OCV	2.02	-	-
C/CafCN	CD@0.8 A g^{-1}	2.13	2.39	2.07
C/KC	CD@0.2 A g^{-1}	2.37	2.31	2.16
C/N-KC	CD@0.2 A g^{-1}	2.10	2.24	2.32
C/CafCN	CV@5 mV s^{-1}	2.27	-	-
